# Supplementary figures and images for: Mutations derived from horseshoe bat ACE2 orthologs enhance ACE2-Fc neutralization of SARS-CoV-2
Source: PLoS Pathog. 2021 Apr 9;17(4):e1009501. doi: 10.1371/journal.ppat.1009501 (PMC8059821; doi:10.1371/journal.ppat.1009501)

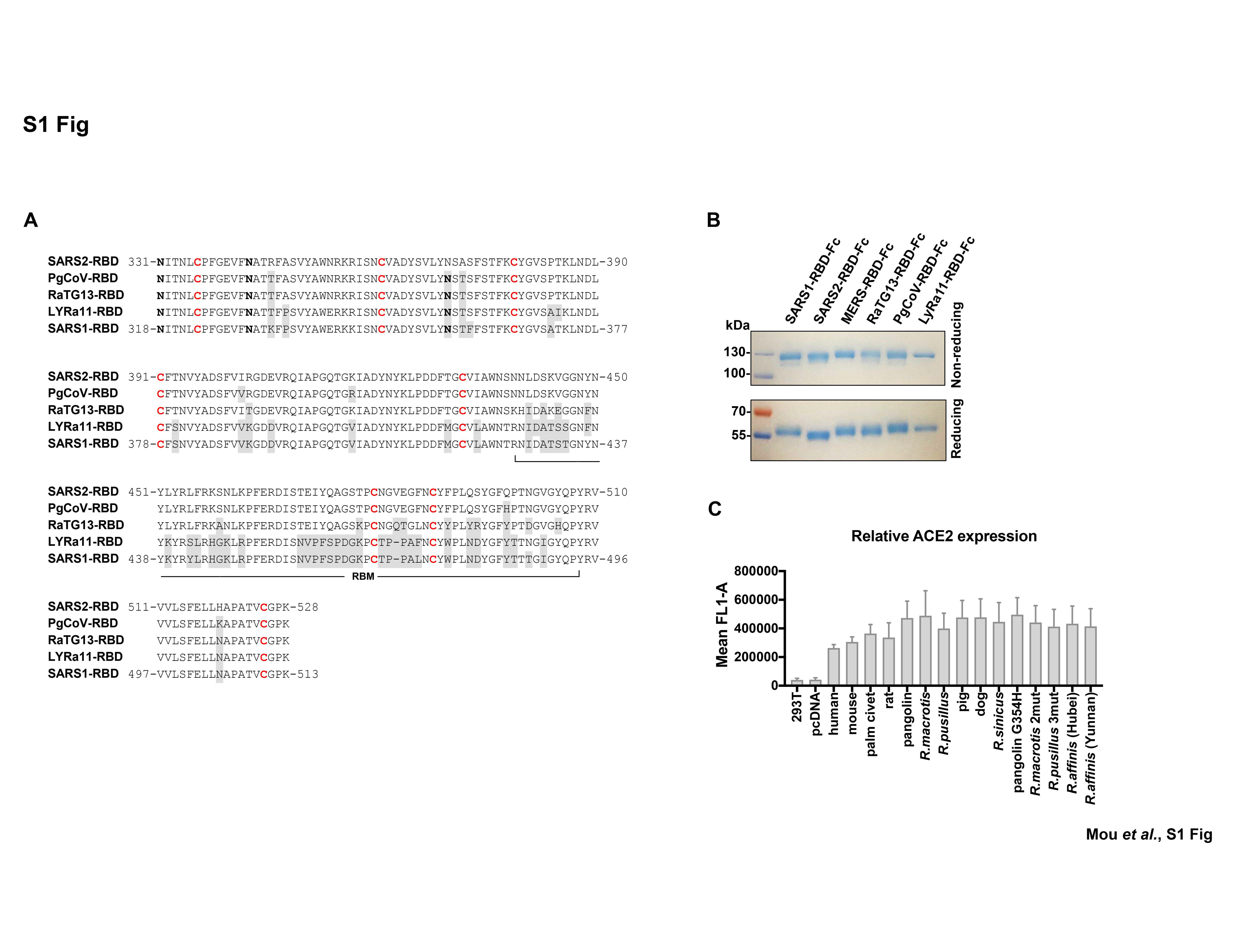

Supplement: S1 Fig — (A) Alignment of amino acid sequences of SARS or SARS-like coronavirus RBDs investigated in this study. Red indicated cysteine residues, bold indicates predicted N-glycosylation site, and grey indicates divergence from the SARS-CoV-2 RBD. The receptor-binding motif (RBM) region that directly contacts human ACE2 is indicated with a bracket. (B) Analysis of purified RBD-Fc proteins. Two micrograms of purified RBD-Fc proteins were analyzed on Novex 4–20% Tris-Glycine gradient gels under reducing (lower) and non-reducing (upper) conditions and stained with Bio-Safe Coomassie G-250 Stain. Position and sizes of the marker proteins are indicated. (C) HEK293T cells used in Fig 1 were transfected with control plasmid (pcDNA) or with plasmids expressing ACE2 orthologs and stained by anti-myc 9E10 antibody. ACE2 expression was determined by flow cytometry, and bars display the mean fluorescence intensity of stained cells from the three independent experiments as described in Fig 1. (TIF) [file ppat.1009501.s001.tif]

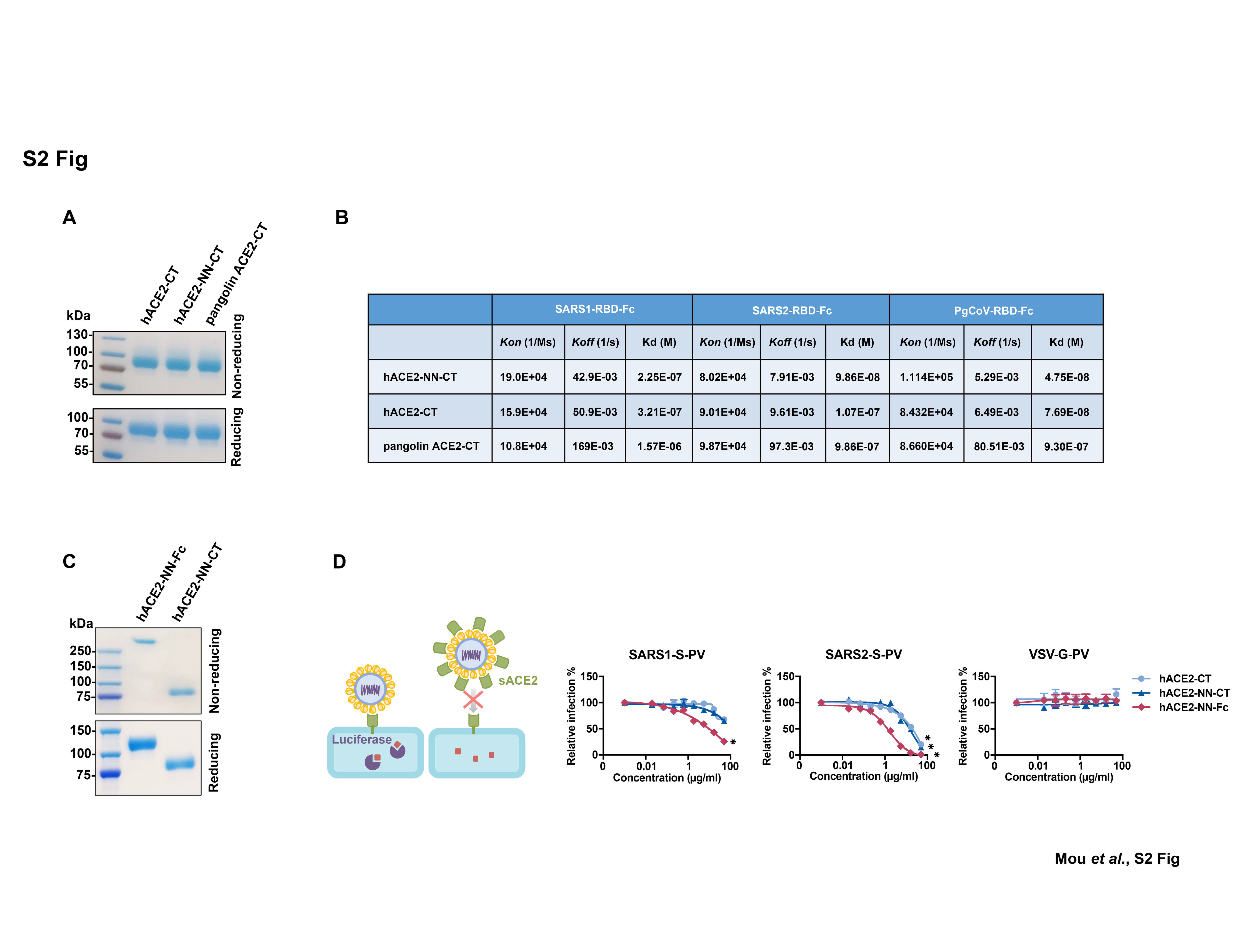

Supplement: S2 Fig — (A) C-terminally tagged (CT) monomeric versions of soluble human ACE2, soluble human ACE2-NN and soluble pangolin ACE2 used in panel B were analyzed as in S1B Fig under non-reducing or reducing conditions as indicated. (B) A summary of numeric values obtained from surface-plasmon resonance studies shown in Fig 2. Soluble monomeric forms of the indicated ACE2 variants were injected for analysis with RBD-Fc variants were captured by an anti-human Fcγ antibody immobilized on a CM5 chip after instantaneous background depletion. Monomeric forms of human ACE2, human ACE2 with its active-site histidines mutated (ACE2-NN), or pangolin ACE2 were injected at five concentrations, serially diluted by two-fold from the highest concentration (100 nM for human ACE2 variants, 500 nM for nonhuman orthologs). (C) C-terminally tagged (CT) versions of soluble human ACE2 and soluble human ACE2-NN-Fc used in panel D were analyzed as in S1B Fig under non-reducing or reducing conditions as indicated. (D) The indicated concentrations of the indicated ACE2-Fc variants were incubated with retroviral pseudoviruses (PV) pseudotyped with the S proteins of SARS-CoV-1 (SARS1-S-PV), SARS-CoV-2 (SAR2-S-PV), or with the G protein of vesicular stomatitis virus (VSV-G-PV). Infection is normalized to that in the absence of inhibitors. Error bars indicate standard error of the mean (S.E.M), and are representative at least two experiments with similar results. Asterisks indicate lack of 95% CI overlap of ACE2 constructs with respect to VSV-G group. (TIF) [file ppat.1009501.s002.tif]

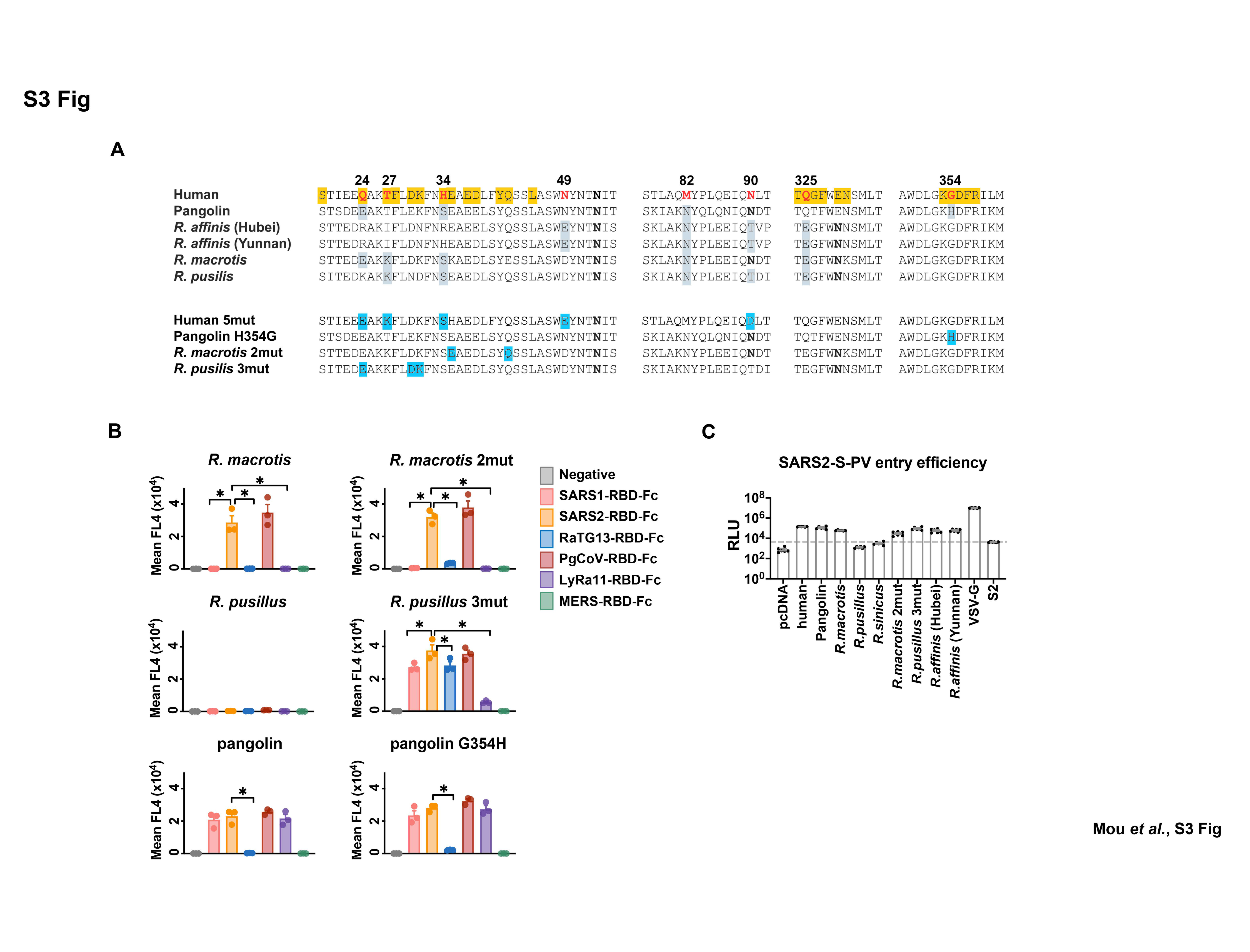

Supplement: S3 Fig — (A) A sequence alignment similar to that in Fig 3B except that three variants in which consensus horseshoe bat residues are introduced, indicated in blue. (B) Studies similar to that in Fig 1 are shown, except that the indicated ACE2 orthologs are compared to variants mutated as shown in panel A. One-way ANOVA was used to test difference across RBDs within each ACE2 ortholog and upon low p value, Dunnett’s multiple comparison test was used to compare each RBD with SARS2-RBD-Fc (*indicates p<0.05). (C) Entry of SARS2-S-PV mediated by transiently expressed human, pangolin, or horse-shoe bat ACE2 orthologs. HEK293T cells were transfected with indicated ACE2 orthologs and then infected with SARS2-S-PV with VSV-G-PV and SARS2-S2-PV as positive and negative controls respectively. Luciferase signal was measured 48 h post transduction, and the data was normalized to the relative expression of ACE2 orthologs. Bars represent averages of at least two independent experiments. Error bars indicate standard error of the mean (S.E.M). (TIF) [file ppat.1009501.s003.tif]

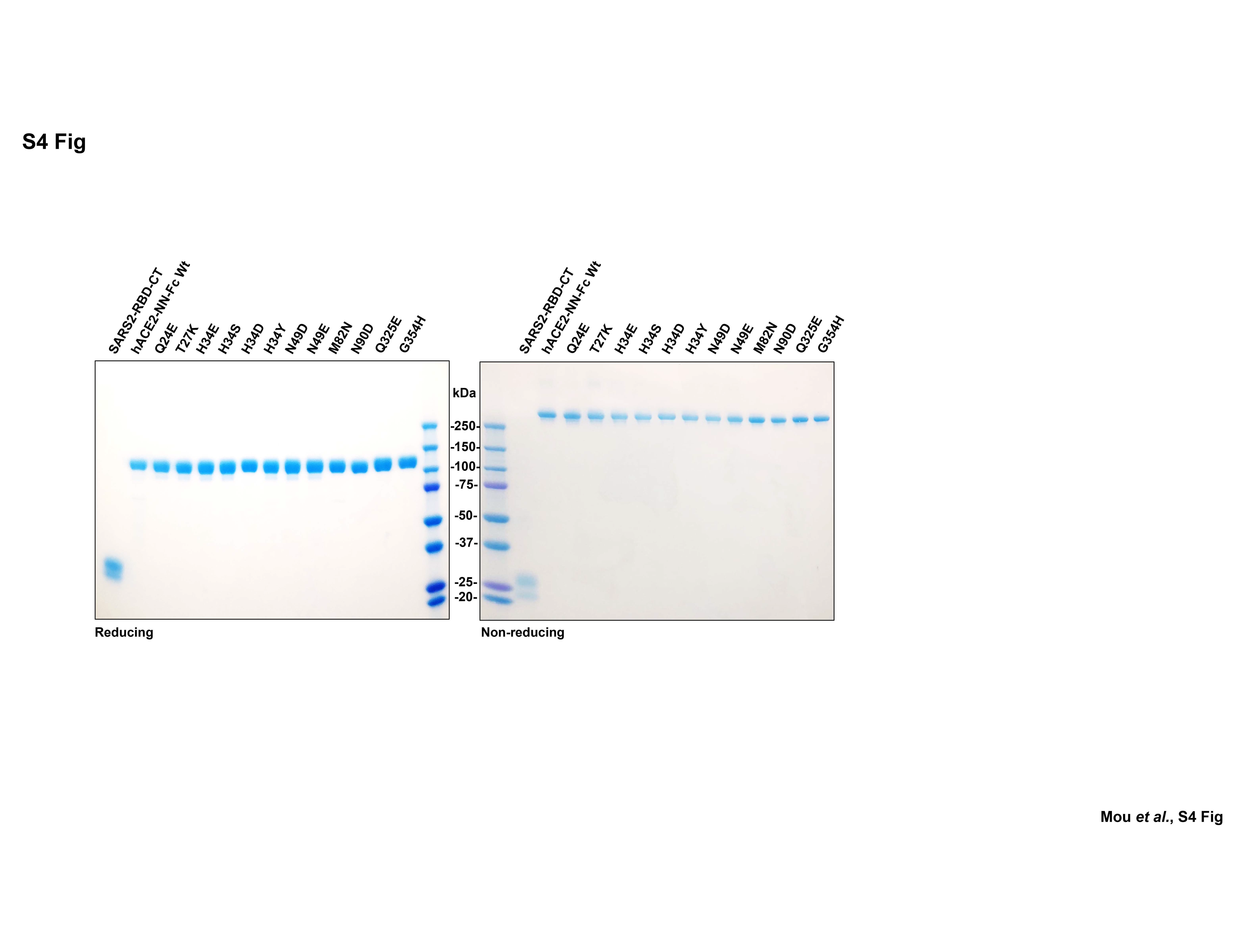

Supplement: S4 Fig — Purified monomeric SARS2-RBD or ACE2-Fc variants including any of the single mutation indicated were analyzed under reducing (left) and non-reducing (right) conditions as described in S1 Fig. (TIF) [file ppat.1009501.s004.tif]

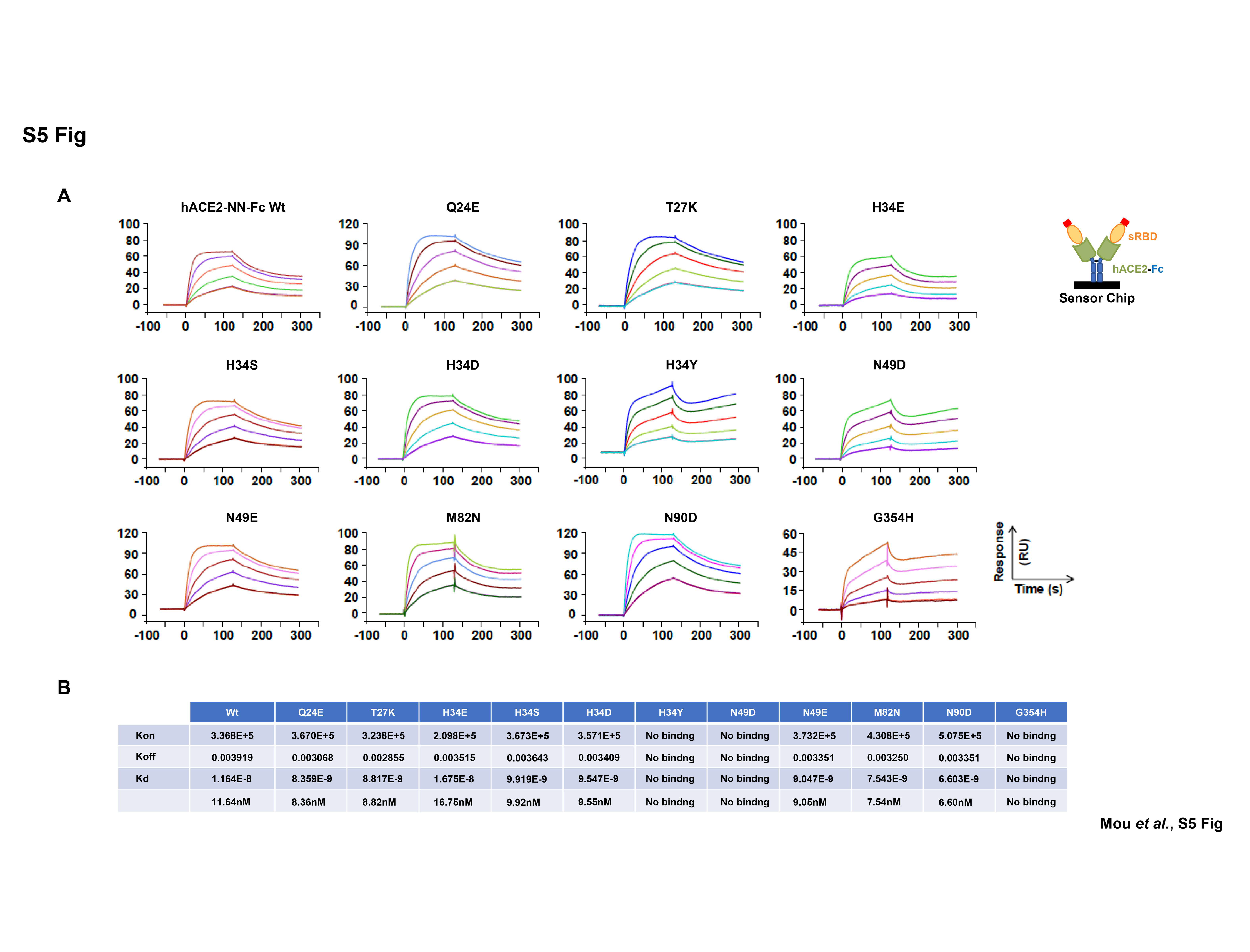

Supplement: S5 Fig — Analysis similar to Fig 2 except that ACE2-Fc variants were captured on the chip and monomeric SARS2-RBD was injected in. (A) Biacore X100 sensorgrams obtained. (B) Numerical values of kon, koff and Kd from the analysis. (TIF) [file ppat.1009501.s005.tif]

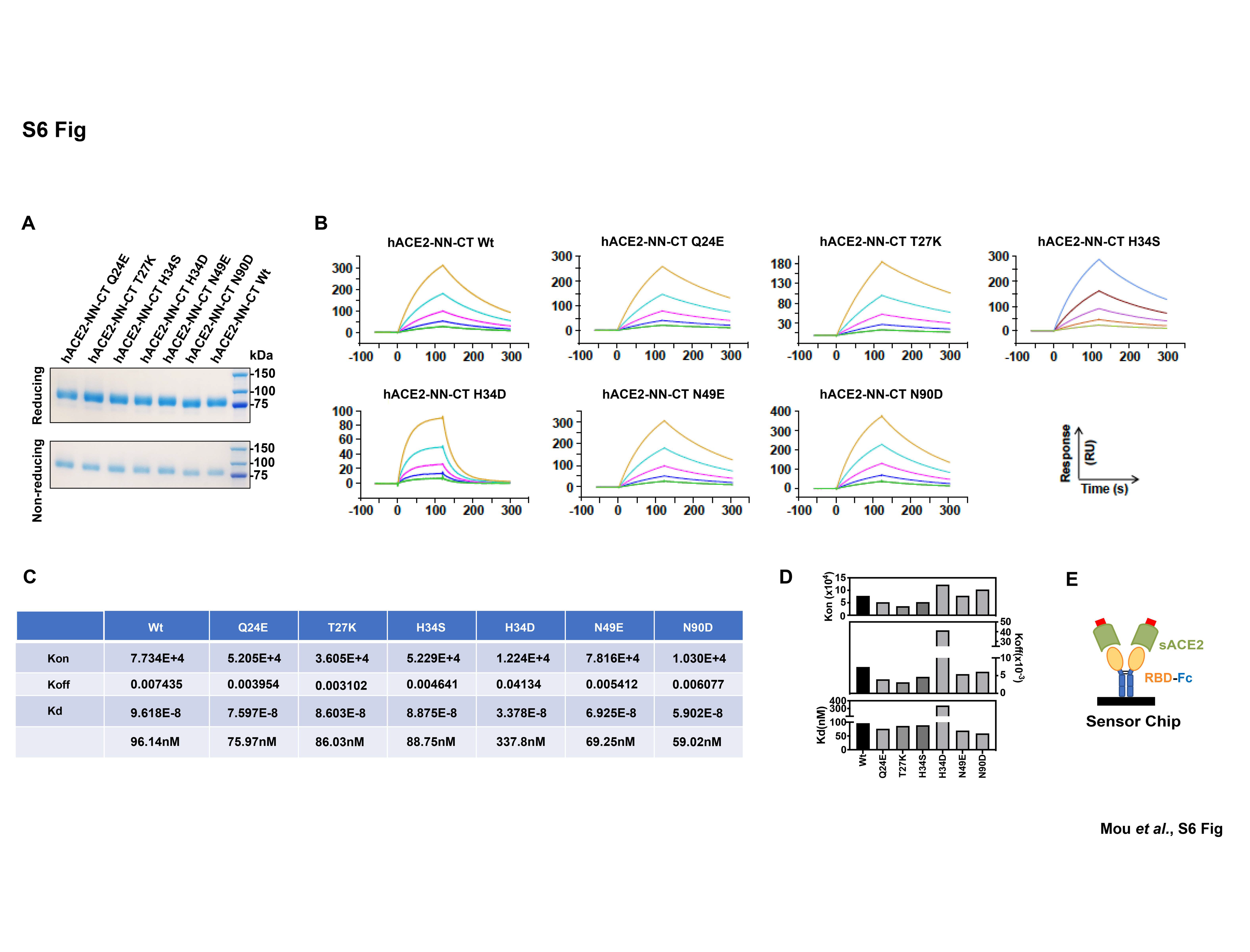

Supplement: S6 Fig — (A) Purified monomeric ACE2 variants were analyzed on 4–20% Tris-Glycine gradient gel under reducing (upper) and non-reducing (lower) conditions as in S1 Fig. (B) The Biacore X100 sensorgrams of the indicated monomeric ACE2 various bound to immobilized SARS2-RBD-Fc are shown. Kon, Koff, and Kd from this analysis that are presented in a table (C) and plotted in (D). (E) A representation of the experiment in (B) indicating that the RBD-Fc was captured and that soluble monomeric ACE2 variants were captured. (TIF) [file ppat.1009501.s006.tif]

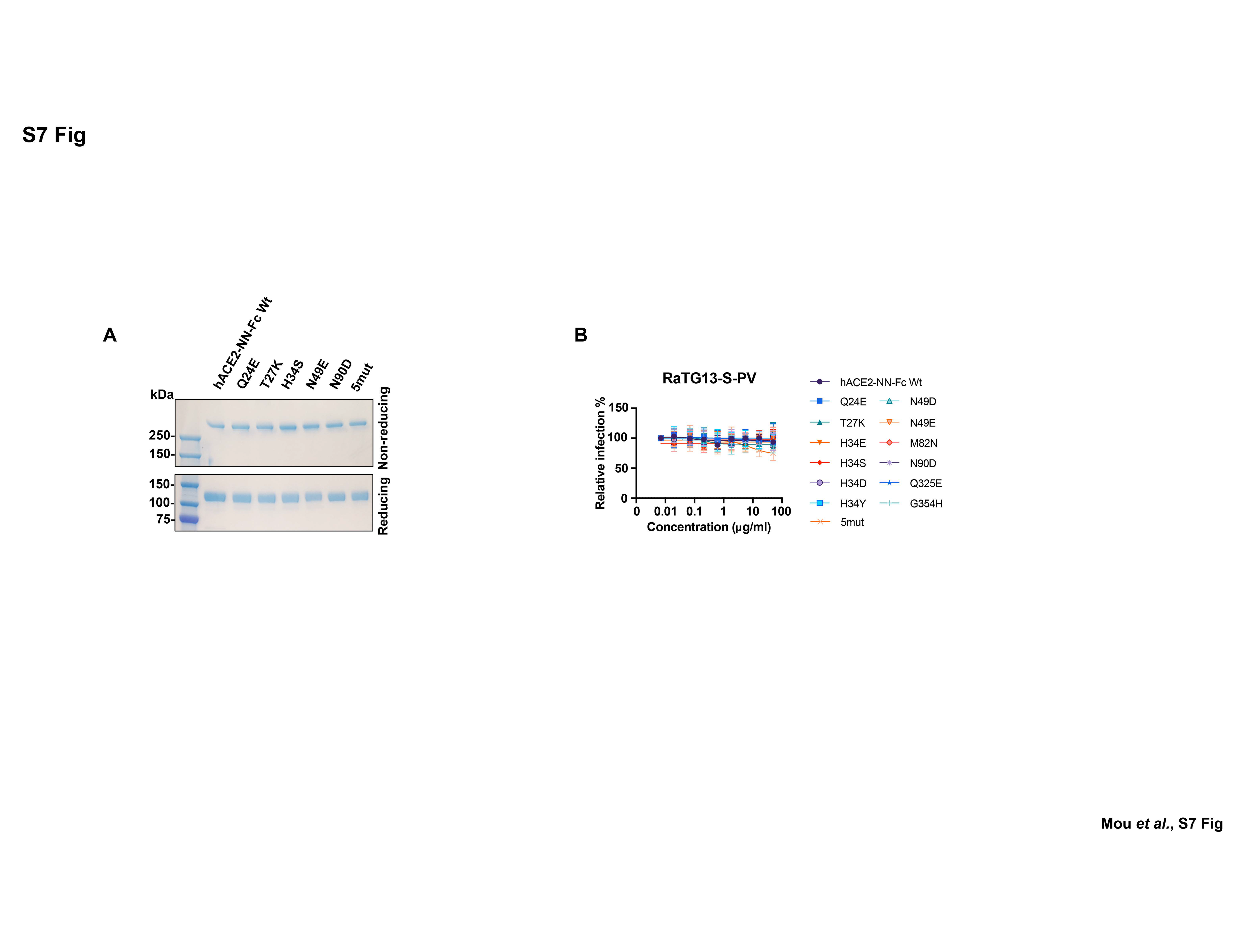

Supplement: S7 Fig — (A) Two micrograms of ACE2-Fc variants including either single mutation or all five mutations were analyzed on Novex 4–20% Tris-Glycine gradient gel under reducing (lower) and non-reducing (upper) conditions. (B) Human ACE2-Fc 5mut demonstrated weak neutralization activity to RaTG13-S-PV. Experiments similar to those shown in Fig 4A except that the neutralization of ACE2-Fc variants to RaTG13-S-PV was directly compared. Relative infection is calculated by dividing luciferase signal values to that in the absence of ACE2-Fc variants. Error bars indicate standard error of the mean (S.E.M). Neutralization data displays at least two independent experiments. (TIF) [file ppat.1009501.s007.tif]

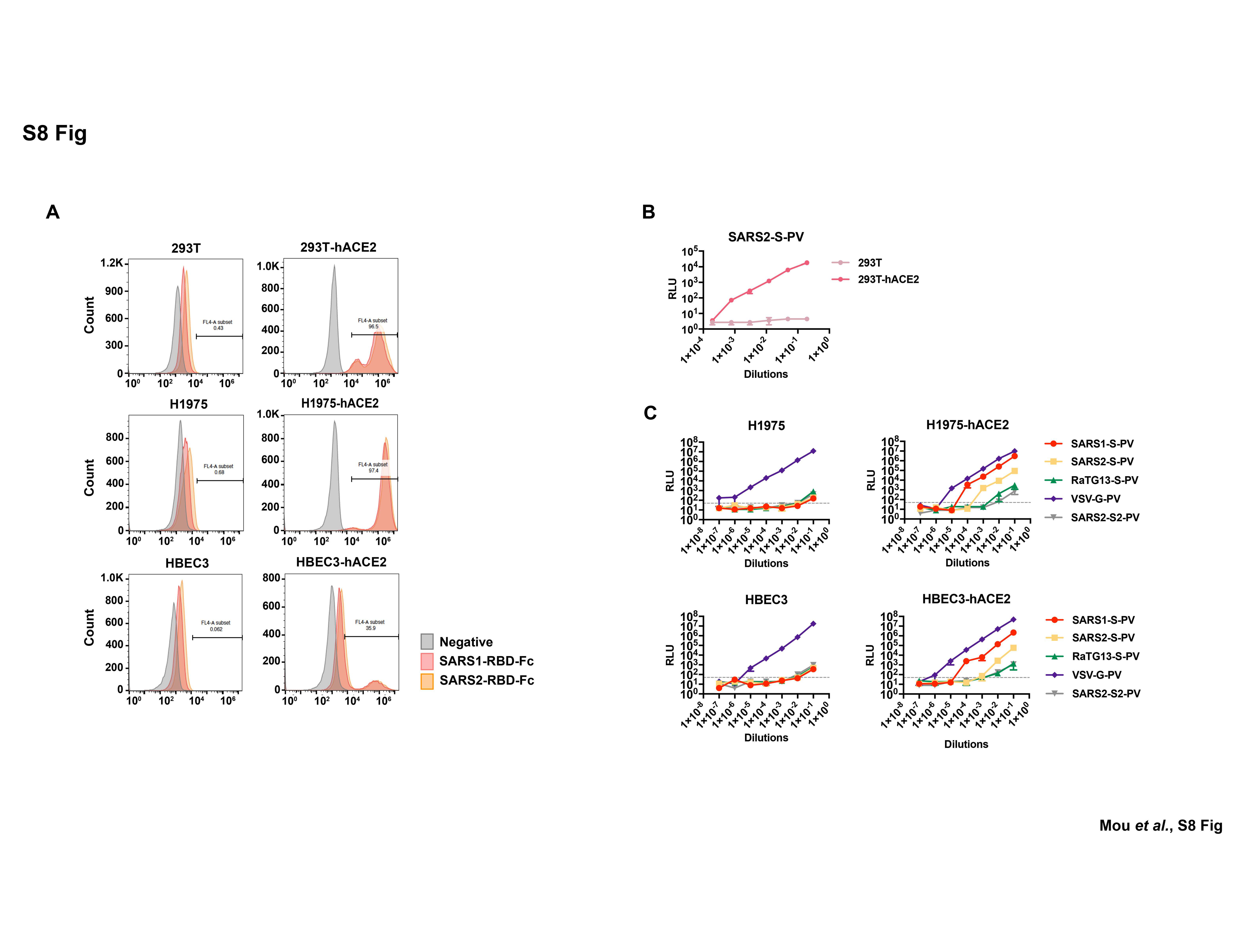

Supplement: S8 Fig — (A) HEK293T cells, NCI-H1975 cells, and HBEC3 cells with and without human ACE2 (hACE2) transduction were stained with 5 ug/ml SARS1-RBD-Fc or SARS2-RBD-Fc, and the binding was detected by goat-anti-human IgG-APC. (B) HEK293T cells with and without hACE2 expression in (A) were transduced with serial-diluted SARS2-S-PV, and the entry efficiency was measured with luciferase signal. (C) Similar experiments were performed with NCI-H1975 cells and HBEC3 cells with and without hACE2 expression, and assayed for transduction with the indicated pseudoviruses. (TIF) [file ppat.1009501.s008.tif]
