# Supplementary material for: Mutations derived from horseshoe bat ACE2 orthologs enhance ACE2-Fc neutralization of SARS-CoV-2
Source: PLoS Pathog. 2021 Apr 9;17(4):e1009501. doi: 10.1371/journal.ppat.1009501 (PMC8059821; doi:10.1371/journal.ppat.1009501)
Supplement: Attachment — Submitted filename: Response_to_Reviewers_final.docx [file ppat.1009501.s010.docx]

**Responses to the Editor and Reviewers**

**Editor’s letter with key scientific comments highlighted**

Dear Dr. Farzan,

Thank you very much for submitting your manuscript "Mutations derived from horseshoe bat ACE2 orthologs enhance ACE2-Fc neutralization of SARS-CoV-2" for consideration at PLOS Pathogens. As with all papers reviewed by the journal, your manuscript was reviewed by members of the editorial board and by several independent reviewers. In light of the reviews (below this email), we would like to invite the resubmission of a significantly-revised version that takes into account the reviewers' comments.

The reviewers mostly agreed that the study has merits in distinguishing between the various horseshoe bat ACE2 orthologs. However, I agree with reviewer #2 that at this point in the epidemic, confirmation of any antiviral activity of ACE2 based reagents must be made in the context of live virus infection, the field is now mature enough that not only live viruses should be used but potentially different isolates that now have mutations in the RBD domains. At the minimum, the key data to confirm that ACE2-Fc orthologs from cognate horseshoe bats are 'potent neutralizers' of SARS-CoV-2 must be performed with relevant live SARS-CoV-2 on susceptible human cells or cell lines. In the absence of live virus data, the claims of this paper does not rise to the standard of advice we require at PLoS Pathogens.

We cannot make any decision about publication until we have seen the revised manuscript and your response to the reviewers' comments. Your revised manuscript is also likely to be sent to reviewers for further evaluation.

When you are ready to resubmit, please upload the following:

[1] A letter containing a detailed list of your responses to the review comments and a description of the changes you have made in the manuscript. Please note while forming your response, if your article is accepted, you may have the opportunity to make the peer review history publicly available. The record will include editor decision letters (with reviews) and your responses to reviewer comments. If eligible, we will contact you to opt in or out.

[2] Two versions of the revised manuscript: one with either highlights or tracked changes denoting where the text has been changed; the other a clean version (uploaded as the manuscript file).

Important additional instructions are given below your reviewer comments.

Please prepare and submit your revised manuscript within 60 days. If you anticipate any delay, please let us know the expected resubmission date by replying to this email. Please note that revised manuscripts received after the 60-day due date may require evaluation and peer review similar to newly submitted manuscripts.

Thank you again for your submission. We hope that our editorial process has been constructive so far, and we welcome your feedback at any time. Please don't hesitate to contact us if you have any questions or comments.

Sincerely,

Benhur Lee
Section Editor
PLOS Pathogens
Benhur Lee
Section Editor
PLOS Pathogens

Kasturi Haldar
Editor-in-Chief
PLOS Pathogens
orcid.org/0000-0001-5065-158X

Michael Malim
Editor-in-Chief
PLOS Pathogens
orcid.org/0000-0002-7699-2064

**Author’s response**

We have addressed the primary concern of the editor by comparing and quantifying neutralization potency of the ACE2-Ig variant 5mut against infectious SARS-CoV-2. These new data are presented in Figures 5G and 5H. As shown, the fold-difference in neutralization potency of 5mut compared with unmodified ACE2-Ig is very similar to that observed with pseudovirus studies.

**Reviewer comments with responses indicated in blue**

**Part I - Summary**
Please use this section to discuss strengths/weaknesses of study, novelty/significance, general execution and scholarship.

Reviewer #1: The manuscript by Mou, Quinlan, Peng and colleagues describes an analysis of the binding affinities of the Receptor Binding Domain (RBD) from several SARS-like coronaviruses with several orthologues of the receptor protein ACE2. Binding affinity is analyzed by different techniques: 1) binding of soluble RBD to cells expressing ACE2 measured by flow cytometry, 2) surface-plasmon resonance (SPR) using both, immobilized RBD versus soluble ACE2 and immobilized ACE2 versus soluble RBD and 3) soluble ACE2 mediated neutralization of infection by pseudo-typed viruses in cell lines expressing ACE2. In general there is good correlation between the results from the different techniques and with previous published results. With the exception of a couple of typos, the manuscript, figures and supporting information are quite clear.

The results show striking differences between the binding affinities of different combinations of RBD-ACE2 which could have implications in the elucidation of the origin and evolution of the SARS-like coronaviruses. Interestingly, the ACE2 orthologues from several bats species of the same genus (suspected to be the original reservoir of the SARS-CoVs ancestors) show very different binding patterns and the authors briefly speculate that it could be indicative of selection pressure on the bat host to limit infection susceptibility.

The results described in this manuscript have 2 potential applications for SARS-CoV2 research/treatment: 1) the description of RBD from animal SARS-like viruses that bind more efficiently to the ACE2 orthologue of laboratory animals (mouse, rat) could contribute to the development of animal models for SARS-CoV2. 2) Introduction of bat-like amino acid changes in the soluble human ACE2 increase binding affinity and neutralization, with a potential application in therapy.

We thank the reviewer for this encouraging comments and have corrected as many typos as we could find.

Reviewer #2: This study by Mou et al describes a detailed investigation of the impact of key mutations of various bat ACE2 molecules on their ability to interact with the RBD of different SARSr-CoVs. The key findings are: a) even for horseshoe bat ACE2 molecules from the same species, there can be important functional difference in the context of RBD binding; b) SARS-CoV and SARS-CoV-2 most likely originated from different horseshoe bat species; c) the SARS-CoV-2 RBD/human ACE2 interaction can still be improved indicating it is not at optimal status yet; d) the human ACE2-Fc incorporating several consensus horseshoe bat ACE2 residues increased its potency to neutralize SARS-CoV-2 PV and can potentially replace potent mAbs as SARS-CoV-2 therapeutics.

We agree with this description of our findings.

**Part II – Major Issues: Key Experiments Required for Acceptance**
Please use this section to detail the key new experiments or modifications of existing experiments that should be absolutely required to validate study conclusions.Generally, there should be no more than 3 such required experiments or major modifications for a "Major Revision" recommendation. If more than 3 experiments are necessary to validate the study conclusions, then you are encouraged to recommend "Reject"

Reviewer #1: The experimental approach is adequate and the conclusions are well supported by the results, without over interpretation of the data. I don’t see the need for additional experiments.

Thank you.

Reviewer #2: Overall, the study is well conducted and some of the findings are of significance to the ongoing research in the context of the “special” relationship between horseshoe bats and SARSr-CoVs. However, the following issues/aspects need to be addressed to improve the current study.

1) Despite the recognised importance of ACE2 in SARSr-CoV infection and emergence, there is no conclusive data to suggest that ACE2 is the only/major entry receptor for all SARSr-CoVs in bats. In fact, the first group of SARS-like-CoVs reported in 2005 (by Li et al) contain Spike proteins which failed to bind ACE2 molecules from the bat species the sequences were derived from.

The reviewer is correct. A number of SARS-like viruses isolated from bats do not utilize any ACE2 ortholog. However, viruses that do not use ACE2 all have a characteristic deletion in their S-protein receptor-binding domains, and specifically in their receptor-binding motifs (RBM). We have noted this through the following sentence in the introduction (lines 101-103) : “*Although some SARS-like viruses have deletions in their RBDs, precluding their use of ACE2, other SARS-like viruses, like SARS-CoV-1 and -2, have intact RBDs and retain their ability to bind and enter cells through ACE2.*” All of the S proteins and viruses characterized here utilize human or bat ACE2.

2) In the same context, the authors need to discuss the limitation of the current study (in fact all published studies on bat ACE2 molecules), i.e., all of the studies were exclusively carried out in the human cell environment. We therefore can not be certain that the findings will be the same if the more relevant bat cells are used in such studies.

The reviewer makes a good point: all entry studies utilized human cells, and subtle differences the cellular context between human and bat might alter infection efficiency. However, much of this study builds on cell-free biophysical studies of the association between ACE2 orthologs and the S protein or its RBD, and results from these studies nicely correlate with our entry studies. We also know from multiple examples, including viruses now emerging in humans, that the affinity between RBD and ACE2 is a major determinant of entry efficiency. We nonetheless have muted our conclusions (e.g lines 270 and 280-281) regarding the origin of the virus to address the uncertainty the reviewer highlights.

3) Despite the usefulness and obvious advantage of no need for BSL3 containment for the PV system used exclusively in this study, I am disappointed not seeing any validation at all using live virus for the current study. There have been reported discrepancies between PV and live virus for SARSr-CoV investigation. I would strongly recommend validation for a few key findings. Especially for the “potent neutralization of SARS-CoV-2” aspect in the context of promoting the mutated human ACE2-Fc as potential therapeutics.

We agree and of now provided neutralization studies of using infectious SARS-CoV-2 (Fig. 5G and H).

4) The authors claimed the mutated human ACE2-Fc has equal or better neutralizing potency as some of the best mAbs. I would like to see side-by-side comparison data on live virus to support such a claim.

We have removed this claim in the discussion (line 314). The potency of ACE2-Ig is lower than many antibodies now reported in the literature. Note however that ACE2-Ig is uniquely equipped to address future emergence of SARS-like viruses, or later iterations of SARS-CoV-2.

**Part III – Minor Issues: Editorial and Data Presentation Modifications**
Please use this section for editorial suggestions as well as relatively minor modifications of existing data that would enhance clarity.

Reviewer #1: The manuscript has a couple of minor errors that should be corrected:
Line 34 (abstract) “closely related SARS-CoV-2,” should say “closely related to SARS-CoV-2,”
Line 51 (author summary) “by interacting the cellular receptor” should be “by interacting with the cellular receptor”
Line 52 (author summary) “is mediated the viral spike” should be “is mediated by the viral spike”
Line 54 (author summary) “with the binding of affinity of” should be “with the binding affinity of”
Line 486 (Figure 2 legend): “were performed the indicated RBD-Fc” should be “were performed with the indicated RBD-Fc”

These typos have been corrected.

Reviewer #2: Lines 34-35 in Abstract: Please replace “RaTG13 …. has been isolated from …” to “RaTG13 …. has been detected from …”. Same for the rest of the paper, please avoid the use of “isolate or isolated” for genome sequences detected in bats. These should be reserved for those with live virus isolation only. This is generating a lot of media confusion: unfortunately, not many journalists can tell the BIG difference between isolation of live virus and detection of genetic sequences in bats. We should hence try our best to reduce such confusion in our scientific publications.

We removed any reference to isolation of viruses, replacing “isolated from” with “obtained from” or “detected in“.

PLOS authors have the option to publish the peer review history of their article ([what does this mean?](https://journals.plos.org/plospathogens/s/editorial-and-peer-review-process#loc-peer-review-history)). If published, this will include your full peer review and any attached files.


If you choose “no”, your identity will remain anonymous but your review may still be made public.


**Do you want your identity to be public for this peer review?** For information about this choice, including consent withdrawal, please see our [Privacy Policy](https://www.plos.org/privacy-policy).

Reviewer #1: No

Reviewer #2: No

Figure Files:

While revising your submission, please upload your figure files to the Preflight Analysis and Conversion Engine (PACE) digital diagnostic tool, [https://pacev2.apexcovantage.com](https://pacev2.apexcovantage.com/). PACE helps ensure that figures meet PLOS requirements. To use PACE, you must first register as a user. Then, login and navigate to the UPLOAD tab, where you will find detailed instructions on how to use the tool. If you encounter any issues or have any questions when using PACE, please email us at [figures@plos.org](mailto:figures@plos.org).

Data Requirements:

Please note that, as a condition of publication, PLOS' data policy requires that you make available all data used to draw the conclusions outlined in your manuscript. Data must be deposited in an appropriate repository, included within the body of the manuscript, or uploaded as supporting information. This includes all numerical values that were used to generate graphs, histograms etc.. For an example see here on PLOS Biology: <http://www.plosbiology.org/article/info%3Adoi%2F10.1371%2Fjournal.pbio.1001908#s5>.

Reproducibility:

To enhance the reproducibility of your results, PLOS recommends that you deposit laboratory protocols in protocols.io, where a protocol can be assigned its own identifier (DOI) such that it can be cited independently in the future. For instructions see [http://journals.plos.org/plospathogens/s/submission-guidelines#loc-materials-and-methods](http://journals.plos.org/plospathogens/s/submission-guidelines)
